# Immunomodulatory activities of isolated compounds from the root-bark of *Cussonia arborea*

**DOI:** 10.1080/13880209.2017.1400078

**Published:** 2017-11-15

**Authors:** Abdulkabir Oladele Oladimeji, Ibrahim Adebayo Oladosu, Almas Jabeen, Aisha Faheem, Mohammed Ahmed Mesaik, Muhammad Shaiq Ali

**Affiliations:** a Natural Products Chemistry Laboratory, Industrial Chemistry Unit, Department of Chemical Sciences, Ondo State University of Science and Technology, Okitipupa, Nigeria;; b H. E. J. Research Institute of Chemistry, International Center for Chemical and Biological Sciences (ICCBS), University of Karachi, Karachi, Pakistan;; c Organic and Medicinal Unit, Department of Chemistry, University of Ibadan, Oyo State, Nigeria;; d Dr Panjwani Center for Molecular Medicine and Drug Research, ICCBS, University of Karachi, Karachi, Pakistan;; e Faculty of Medicine, University of Tabuk (UT), Tabuk, Saudi Arabia

**Keywords:** Araliaceae, T-cell proliferation assay, reactive oxygen species, triterpenoids, oleanolic acid, hederagenin, stigmasterol, NMR spectroscopy, steroid

## Abstract

**Context:***Cussonia arborea* Hochst. ex A. Rich (Araliaceae) is a folk medicine used to treat various diseases. However, there is no report of the root phytochemistry.

**Objective:** This study isolates and identifies the immunomodulatory compounds from root-bark of *C. arborea*.

**Materials and methods:** The methanol extract (18 g) was subjected to repeated column chromatography resulting in isolation of five compounds (**1**–**5**). Structure determination was achieved by analysis of their 1 D and 2 D NMR, and mass spectroscopy. The compounds (100–1.0 μg/mL) were examined immunomodulatory for effect on production of reactive oxygen species (ROS) from whole blood phagocytes and on proliferation of T-cells. The compounds cytotoxicity (100–1.0 μg/mL) was evaluated on NIH-3T3 normal fibroblast cells.

**Results:** Three pentacyclic triterpenoids [3, 23-dihydroxy-12-oleanen-28-oic acid (**1**), 3β-hydroxylolean-12-en-28-oic (**2**) and 23-hydoxy-oxo-urs-12-en-28-oic acid (**5**)], two phytosterols: [stigmasterol (**3**)] and [3-*O*-β-d-glucopyranosyl stigmasterol (**4**)] were all isolated from the methanol soluble extract. All the tested compounds (**1**–**4**) were found to be nontoxic on NIH-3T3 cells. Compound **1** and **2** moderately inhibited the production of ROS (IC_50_ = 24.4 ± 4.3 and 37.5 ± 0.1 μg/mL, respectively) whereas compound **2** exhibited the highest inhibitory effect (IC_50_ = 12.6 ± 0.4 μg/mL) on proliferation of phytoheamagglutinin (PHA) activated T-cells.

**Conclusions:** The isolated compounds (**1**–**5**) are reported for the first time from this species. In addition, compound **2** with suppressive potential on production of intracellular ROS and proliferation of T-cells could be of immense value in control of autoimmune diseases as well as in immune compromised patients.

## Introduction

The genus *Cussonia* (Araliaceae) is well known in folk medicines for the treatment of malaria, mental illness, eye problems, sexually transmitted diseases, skin problems, cancer, wounds, etc. (Kougan et al. 2009; De Villiers et al. 2010). They are also widely used for the treatment of rheumatism and dysmenorrhea (Dubois et al. 1986). *Cussonia arborea* Hochst. ex A. Rich is a medium sized deciduous tree with rough and corky bark and has a wide distribution in Africa, from western into the central and eastern areas of Africa. It is known as ‘Sigo’ among the Yorubas of south-western Nigeria where the leaves are used, mainly for the treatment of painful menstruation, biliousness, allergic reactions, constipation and epilepsy (Ogunlesi et al. 2008). Triterpene glycosides, arboreasides A–E, ciwujianoside C_3_ and 28-*O*-α-L-rhamnosyl-(1 → 4)-β-d-glucopyranosyl-(1 → 6)-β-d-glucopyranoside of 23-hydroxyursolic acid only, were reported to have been isolated from the stem bark of this species (Kougan et al. 2009). A literature survey showed that no significant chemical and biological work has been done on other parts of *C. arborea.* In this paper, for the first time, we report the isolation and structure elucidation of 5 compounds: 3, 23-dihydroxy-12-oleanen-28-oic acid (**1**) 3β-hydroxylolean-12-en-28-oic, (**2**) stigmasterol, (**3**) 3-*O*-β-d-glucopyranosyl stigmasterol, (**4**) and 23-hydroxy-3-oxo-urs-12-en-28-oic acid and (**5**) from the root-bark of this plant. In present study, we examined the immunomodulatory activities of the isolated compounds (**1**–**4**) using two different parameters of innate and adaptive immune responses, that is, effect on production of intracellular reactive oxygen species (ROS) from zymosan activated whole blood phagocytes and on proliferation of phytoheamagglutinin (PHA) activated T-lymphocytes to evaluate their potential for the control of harmful immune responses. The cytotoxicity of compounds was evaluated on NIH-3T3 fibroblast by MTT (3-(4,5-dimethylthiazol-2-yl)-2,5-diphenyltetrazolium bromide) assay. This is the first report of immunomodulatory study of *C. arborea.*

## Materials and methods

### Experimental

The nuclear magnetic resonance (NMR) spectra were recorded in deuterated chloroform or methanol or pyridine on a Bruker Avance 500 and 400 MHz NMR spectrometers. IR spectra were recorded using JASCO 302-A spectrometer, respectively. High resolution electron ionized mass spectrometry (HR EIMS) and fast atomic bombardment mass spectrometry (FABMS) were carried out using MAT 95XP and JMS HX-110 mass spectrometers, respectively. All reagents used were of analytical grade.

### Plant material

The whole plant of *C. arborea* was collected in May, 2012, from a farm Land at Eruwa in Ibarapa East Local Government area of Oyo state, Nigeria. The root-barks were air-dried at room temperature and pulverized. The voucher specimen (UIH-22340) was deposited at the Herbarium unit of Botany and Microbiology Department, University of Ibadan, Nigeria after identification and authentication by Mr. D. O. Esimekhuai.

### Extraction, isolation and characterization

The air dried *C. arborea* root-bark (1 kg) was extracted with methanol (3 × 2.5 L) for two weeks at room temperature (20–25 °C). The combined extracts were concentrated under pressure using rotary evaporator, preset at 37 °C yielding a light greenish powder (18 g). The methanol extract (18 g) was pre-adsorbed on silica gel and introduced to column (length = 82 cm, internal diameter = 5.0 cm) packed with silica gel as stationary phase. The solvent system used in eluting the column are hexane (100%, 1000 mL), hexane:EtOAc (19:1, 9:1, 17:3, 4:1, 3:1, 7:3, 13:7, 3:2, 11:9, 1:1, 2:3, 3:7, 1:4, 1:9, 1000 mL each), EtOAc (100%, 1000 mL), EtOAc:MeOH (19:1, 9:1, 1000 mL each), successively. Collection volume of eluent employed was 1000 mL/vial. A total of 80 fractions were collected (F_1_–F_80_). Fraction F_10_ and F_12_ eluted with hexane:EtOAc 17:3 and 7:3 afforded compounds **3** (8.0 mg) and **2** (22.0 mg), respectively, as white solids. Fraction F_25_ eluted with hexane:EtOAc (1:1) was concentrated under reduced pressure to yield a white precipitate amorphous powder (250 mg). The precipitate was then collected by filtration and purified by recrystallisation from MeOH by the slow evaporation of the solvent at room temperature to give the compound **1** (150.0 mg) as pure colourless crystal. Compound **4**, a white amorphous compound, was also obtained from fraction F_37_, which was eluted with 80% EtOAc in hexane. Fraction F_37_ was re-subjected to silica gel Column Chromatography eluted with mixture of dichloromethane and methanol (97:3) to afford compound **5** (5.0 mg).

### Identification of isolated compounds 1–5

**Figure 1.**
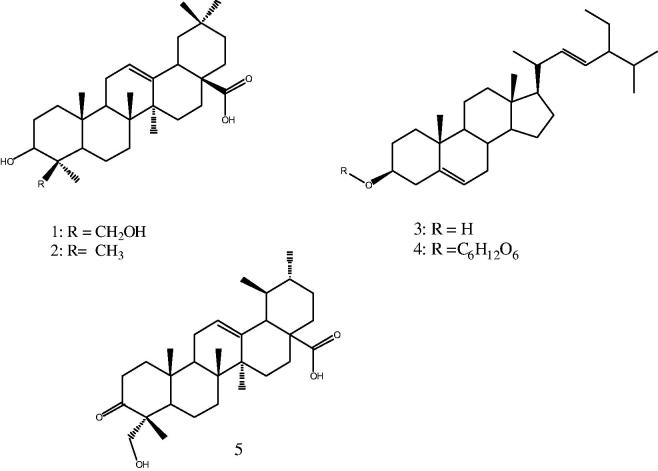
Chemical structures of isolated compounds from *C. arborea*.

The structures of compounds **1**–**5** (Figure 1) were elucidated by extensive spectroscopic measurements and by comparison with data in the literature (Mahato and Kundu 1994; He et al. 2003; Tapondjou et al. 2003; Forgo and Kövér 2004; Senthilkumar and Reetha 2011).

#### *3, 23-Dihydroxy-12-oleanen-28-oic acid* (1)

Melting point: 292 °C; Colourless crystalline solid; IR(KBr): 3450 (OH), 2943, 1699 (C = O), 1463, 1038 cm^−1^; EI-MS: *m/z* (rel. int): 472 (M^+^, 6), 454 (7), 396 (7), 248 (100), 223 (23), 203 (86), 175 (69), 161 (9), (45), 119 (25); HREIMS: 472.3547 (Calcd. 472.3554 for C_30_H_48_O_4_). ^1^H-NMR: (CD_3_OD, 400 MHz): δ 0.69 (3H, s, H-24), 0.81 (3H, s, H-26), 0.90 (3H, s, H-29), 0.93 (3H, s, H-30), 0.97 (3H, s, H-25), 1.12 (3H, s, H-27), 2.83 (1H, dd, *J* = 4.0,14 Hz, H-18), 3.50 (1H, d, *J =* 10.8 Hz, H-23), 3.57 (1H, m, H-3), 5.21 (1H, m, H-12). ^13 ^C-NMR (CD_3_OD, 100 MHz): See Table 1.

**Table 1.** ^1^H and ^13 ^C NMR spectra data of Compound **1** (400 and 100 MHz, CD_3_OD) compared with ^13 ^C NMR data of literature.

Position	^1^H δ (multiplicity)	^13^C	^13^C*	DEPT
1.	1.61 (m)	39.47	38.1	CH_2_
2.		25.31	25.3	CH_2_
3.	3.57 (m)	73.78	73.9	CH
4.	–	42.95	43.3	C
5.		48.2	overlapped^a^	CH
6.	1.52 (m)	19.11	19.11	CH_2_
7.		33.46	33.8	CH_2_
8.	–	37.90	37.9	C
9.		overlapped^a^	overlapped^a^	CH
10.	–	37.84	37.8	C
11.		24.36	24.4	CH_2_
12.	5.21 (m)	123.60	123.6	CH
13.	–	143.25	143.3	C
14.	–	43.29	43.0	C
15.		28.82	27.5	CH_2_
16.		24.51	23.9	CH_2_
17.	–	47.62	47.2	C
18.	2.83 (dd)	42.72	42.7	CH
19.		47.22	47.6	CH_2_
20.	–	34.87	33.5	C
21.		33.82	34.9	CH_2_
22.		32.00	33.9	CH_2_
23.	3.50 (d)	67.18	67.3	CH_2_
24.	0.69 (s)	12.74	12.8	CH_3_
25.	0.97 (s)	16.25	16.4	CH_3_
26.	0.81 (s)	17.74	17.8	CH_3_
27.	1.12 (s)	24.04	24.1	CH_3_
28.	–	181.87	181.7	C
29.	0.90 (s)	33.56	33.6	CH_3_
30.	0.93 (s)	21.58	21.6	CH_3_

* He et al. (2003).

a overlapped with signals of MeOD.

#### *3β-Hdroxylolean-12-en-28-oic* (2)

Melting point: 271–273 °C; White amorphous solid; EI-MS *m/z* (rel. int.): 456 (M^+^, 2), 410 (2), 300 (2), 248 (100), 233 (10), 203 (77), 189 (12), 133 (12), 189 (100), 133 (13), 119 (9); HREIMS: 456.3566 (Calcd. 456.3605 for C_30_H_48_O_3_). ^1^H-NMR (400 MHz, CDCl_3_): δ 0.75 (3H, s, H-24), 0.88 (3H, s, H-29), 0.88 (3H, s, H-25), 1.11 (3H, s, H-27), 0.96 (3H, s, H-23), 0.73 (3H, s, H-26), 0.89 (3H, s, H-30), 1.57 (2H, m, H-19), 2.78 (1H, m, H-18), 3.18 (1H, t, H-3), 5.26 (1H, m, H-12). ^13 ^C-NMR (CDCl_3_, 100 MHz): See Table 2.

**Table 2.** ^1^H and ^13 ^C NMR spectra data of Compound **2** (400 and 100 MHz, ppm in CDCl_3_) compared with ^13 ^C NMR data of literature.

Position	^1^H δ (multiplicity)	^13^C	^13^C*	DEPT
1.	1.57 (m)	38.36	38.5	CH_2_
2.	1.57 (m)	27.14	27.4	CH_2_
3.	3.18 (m)	78.99	78.7	CH
4.	–	38.73	38.7	C
5.	0.7 (m)	55.17	55.2	CH
6.	1.52 (m)	18.26	18.3	CH_2_
7.		32.58	32.6	CH_2_
8.	–	39.20	39.3	C
9.	1.50 (m)	47.59	47.6	CH
10.	–	37.04	37.0	C
11.	1.57 (m)	22.90	23.1	CH_2_
12.	5.26 (m)	122.61	122.1	CH
13.	–	143.55	143.4	C
14.	–	41.55	41.6	C
15.		27.64	27.7	CH_2_
16.	1.85 (m)	23.36	23.4	CH_2_
17.	–	46.48	46.6	C
18.	2.78 (m)	40.96	41.3	CH
19.	1.57 (m)	45.82	45.8	CH_2_
20.	–	30.66	30.6	C
21.		33.75	33.8	CH_2_
22.		32.39	32.3	CH_2_
23.	0.96 (s)	28.07	28.1	CH_3_
24.	0.75 (s)	15.54	15.6	CH_3_
25.	0.88 (s)	15.30	15.3	CH_3_
26.	0.73 (s)	17.09	16.8	CH_3_
27.	1.11 (s)	25.92	26.0	CH_3_
28.	–	183.13	181.0	C
29.	0.88 (s)	33.06	33.1	CH_3_
30.	0.89 (s)	23.55	23.6	CH_3_

* Mahato and Kundu (1994).

#### *Stigmasterol* (3)

Melting point: 168–169 °C; White needle solid; IR (KBr): 1727, 1239, 3060, 1637, 865 cm^−1^; EI-MS: *m/z* (rel. int): 412 (M^+^, 100), 396 (47), 381 (34), 369 (17), 329 (40), 303 (46), 255 (66), 233 (42), 213 (48), 159 (43), 107 (52), 83 (44); HREIMS: 412.3700 (Calcd. 412.3707 for C_29_H_48_O). ^1^H NMR: (CDCl_3_, 400 MHz): δ 0.66 (3H, s, H-18), 0.78 (3H, t, H-29), 0.82 (3H, d, *J* = 7.5 Hz, H-26), 1.01 (3H, d, *J* = 6.5 Hz, H-21), 0.99 (3H, s, H-19), 4.96 (1H, dd, *J* = 15.2, 8.8 Hz, H-23), 5.10 (1H, dd, *J* = 15.2, 8.4 Hz, H-22), 5.32 (1H, m, H-6). ^13 ^C-NMR (CDCl_3_, 100 MHz): See Table 3.

**Table 3.** ^1^H and ^13 ^C NMR spectra data of Compound **3** (400 and 100 MHz, CDCl_3_) compared with ^13 ^C NMR data of literature.

Position	^1^H δ (multiplicity, J)	^13^C	^13^C*	DEPT
1.	1.84, 1.05	37.26	37.6	CH_2_
2.	1.47	31.68	31.9	CH_2_
3.	3.46 (m)	71.81	72.0	CH
4.	2.21 (m)	42.32	42.5	CH_2_
5.		140.76	140.8	C
6.	5.32 (t)	121.70	121.8	CH
7.		31.68	32.1	CH_2_
8.	1.80	31.91	32.2	CH
9.	0.9	50.17	50.5	CH
10.		36.52	36.5	C
11.		21.08	21.2	CH_2_
12.	1.92, 1.13	39.69	40.0	CH_2_
13.		42.22	42.2	C
14.	0.99	56.88	57.1	CH
15.		24.36	24.5	CH_2_
16.	1.67	28.91	28.9	CH_2_
17.	1.33	55.97	56.3	CH
18.		12.05	12.2	CH_3_
19.	0.99	19.40	19.5	CH_3_
20.	1.98	40.48	40.4	CH
21.		21.09	21.4	CH_3_
22.	5.10 (dd, 15.2, 8.4 Hz)	138.31	138.3	CH
23.	4.96 (dd, 15.2, 8.8 Hz)	129.29	129.7	CH
24.	1.51	51.24	51.5	CH
25.		31.91	32.2	CH
26.	0.82	21.21	21.2	CH_3_
27.	0.76	18.98	19.2	CH_3_
28.	1.41, 1.13	25.40	25.4	CH_2_
29.	0.78	12.24	12.2	CH_3_

* Forgo and Kövér (2004).

#### *3-*O*-β-d-Glucopyranosyl stigmasterol* (4)

Melting point: 290–292 °C; White amorphous solid; IR (KBr): 3450, 1727, 1637, 1239, 865 cm^−1^; EI-MS: *m/z* (rel. int): 412 (C_29_H_48_O, aglycone, 25), 394 (100), 381 (23), 369 (9), 351 (32), 255 (80), 213 (36), 159 (43), 147 (50), 83 (72); FABMS (−ve) = 573.3674[M–H]^−^ (Calcd. 574.4235 for C_35_H_58_O_6_). ^1^H NMR: (C_5_D_5_N, 400 MHz,): δ 0.64 (3H, s, H-18), 0.88 (3H, t, H-29), 0.86 (3H, d, *J* = 6.2 Hz, H-26), 1.06 (3H, d, *J* = 6.5 Hz, H-21), 0.93 (3H, s, H-19), 5.02 (1H, dd, *J* = 15.2, 6.4 Hz, H-23), 5.18 (1H, dd, *J* = 15.2, 6.4 Hz, H-22), 5.34 (1H, br, s, H-6), 5.05 (1H, d, *J* = 7.8 Hz, H-1′) ^13 ^C-NMR: (C_5_D_5_N, 100 MHz): See Table 4.

**Table 4.** ^1^H and ^13 ^C NMR spectra data of Compound **4 spectra** (400 and 100 MHz, C_5_D_5_N).

Position	^1^H δ (multiplicity, J)	^13^C	DEPT
1.	1.72	37.50	CH_2_
2.	1.47	32.12	CH_2_
3.	4.28 (m)	72.97	CH
4.	2.4 (m)	39.36	CH_2_
5.		140.94	C
6.	5.34 (t)	121.93	CH
7.		32.10	CH_2_
8.	1.3	32.07	CH
9.	0.88	50.37	CH
10.		36.96	C
11.		22.94	CH_2_
12.	1.92, 1.13	39.96	CH_2_
13.		42.37	C
14.	0.99	56.94	CH
15.		24.56	CH_2_
16.	1.67	29.34	CH_2_
17.	1.33	56.09	CH
18.	0.64	12.16	CH_3_
19.	0.93	19.44	CH_3_
20.	2.00	40.80	CH
21.	1.06	21.49	CH_3_
22.	5.18 (dd, 15.2, 8.4 Hz)	138.85	CH
23.	5.02 (dd, 15.2, 8.8 Hz)	129.49	CH
24.	1.50	51.44	CH
25.		32.91	CH
26.	0.86	19.20	CH_3_
27.	0.76	14.26	CH_3_
28.	1.25	25.72	CH_2_
29.	0.88	12.54	CH_3_
1′	5.05 (d, 7.8 Hz)	102.59	CH
2′	3.92	78.10	CH
3′	4.25	78.64	CH
4′	4.04	75.37	CH
5′	4.25	78.52	CH
6′	4.28	71.72	CH_2_

#### *23-Hydroxy-3-oxo-urs-12-en-28-oic acid* (5)

Melting point: 170–172 °C; Colourless solid; IR (KBr): 3453 (OH), 2944, 1699 (C = O), 1463, 1384, 1038 cm^−1^; EI-MS: *m/z* (rel. int): 470 (M^+^, 2), 426 (9), 424 (5), 248 (100), 203 (73), 189 (32), 175 (24), 133 (85), 119 (34), 91 (25), 81 (35), 71 (40), 57 (66), 44 (57). HREIMS: 470.3421 (Calcd. 470.3398 for C_30_H_46_O_4_). ^1^H NMR: (CDCl_3_, 500 MHz): δ 0.77 (3H, s, H-24), 0.83 (3H, d, *J* = 6.5 Hz, H-29), 0.87 (3H, s, H-25), 0.92 (3H, d, *J* = 6.5 Hz, H-30), 1.06 (3H, s, H-26), 1.23 (3H, s, H-26), 2.15 (1H, d, *J* = 11.5 Hz, H-18), 3.40 (1H, d, *J* *=* 10.5 Hz, H-23a), 3.70 (1H, d, *J* *=* 10.5 Hz, H-23 b), 5.23 (1H, b, s, H-12).

### Biological studies

All studies on human blood samples were carried out after an approval from independent ethics committee, International Center for Chemical and Biological Sciences, University of Karachi (ICCBS, UoK), No: ICCBS/IEC-008-BC-2015/Protocol/1.0, and with written informed consent from the volunteers. In this study, the blood used for both oxidative burst and lymphocyte proliferation test was from same volunteer.

### Oxidative burst assay

#### Preparation of serum opsonized zymosan (SOZ)

Exact 10 mL of 0.3% SOZ was prepared by adding 30 mg of zymosan powder [Fluka, Buchs, Switzerland] in 2 mL of pooled human serum, then volume was made by adding 8 mL of Tris base NaCl. The mixture was vortexed and incubated in shaking water bath at 37 °C for 30 min then centrifuged at 2000 *g* for 5 min at room temperature. Supernatant was discarded and pellet was resuspended in 10 mL of Trisbase NaCl. Final concentration of 0.075% SOZ was achieved in each experimental well.

#### Preparation of luminol

Luminol solution 7 × 10^−5 ^M (10 mL) was prepared by dissolving 1.8 mg of luminol [Research Organics, Cleveland, OH, USA] in 1 mL of borate buffer. 9.0 mg of gelatin was then dissolved in 9.0 mL of HBSS^++^ (Hanks Balanced Salt Solution, containing calcium chloride and magnesium chloride) [Sigma, St. Louis, MO, USA] then the mixture of luminol + borate buffer was transferred in it. Final concentration of 1.7 × 10^−5 ^M was achieved in each experimental well. The assay was performed on heparinized human whole blood collected from healthy volunteer. The blood was diluted in HBSS^++^ with a dilution of 1:50. SOZ was used as an activator. As phagocytes have receptors for SOZ on their surface so they are the major producer of ROS in activated cells. Luminol-enhanced chemiluminescence assay was performed, as described by Helfand et al. (1982) with some modifications. Briefly 25 µL of 1:50 diluted whole blood was incubated with 25 µL of three different concentrations of compounds (1, 10 and 100 µg/mL), each in triplicate. Control wells received HBSS^++^ and cells, but no compounds. Test was performed in white half area 96 well plates [Costar, NY, USA]. The plates were incubated at 37 °C for 15 min in the thermostat chamber of luminometer [Labsystems, Helsinki, Finland]. After incubation, 25 µL of 0.3% SOZ and 25 µL of 7 × 10^−5 ^M luminol were added into each well, except blank wells (containing only HBSS^++^). Results were monitored as relative light units (RLU) reading, with peak and total integral values set with repeated scans at 50 s intervals, and 1 s point of measuring time. Results were collected as separate graph for each well showing peak value, peak time of cell activity and total integral. The drug Ibuprofen was used as standard.

#### Lymphocyte proliferation assay

Lymphocytes were isolated from human heparinized blood. Briefly 10 mL of blood was aseptically collected in heparin containing tube from healthy volunteer. Blood was then mixed with equal volume (1:1) of incomplete RPMI (Roswell Park Memorial Institute) media in 50 mL sterile falcon tube. Lymphocyte separation media (LSM) (5 mL) was added into two sterile 15 mL falcon tubes and 10 mL of the mixture containing blood and RPMI was gently layered on LSM in each tube. Tubes were then centrifuged at 400 *g* for 20 min at room temperature. The buffy layer appeared at the interface of LSM and plasma was carefully collected and washed at 4 °C for 10 min at 300 *g*. The supernatant was discarded and pellet containing peripheral blood mononuclear cells (PBMNCs) was collected. Cell viability and counting was performed using trypan blue dye.

^3^H-Thymidine incorporated T-cell proliferation assay was performed as described by Mesaik et al. (2009). Briefly three different concentrations (1, 10 and 100 μg/mL) of test compounds were added in the white 96 wells round bottom tissue culture plate using RPMI supplemented with 5% foetal calf serum (v/v) (5% FCS/RPMI) as a diluent to a final volume of 100 μL in each well in triplicates. After that 50 μL of isolated PBMNCs were added at a concentration of (2 × 10^6^ cell/mL). Cells were then stimulated by adding 50 μL of 7.5 μg/mL phytoheamagglutinin-L (PHA-L) which acts as mitogen only for T-cells among other PBMNCs. The final volume of 200 μL was achieved in each well. The wells received 50 μL cells and 150 μL of 5% FCS/RPMI served as negative control where as positive control contains 50 μL cells, 50 μL of PHA-L and 100 μL of 5% FCS/RPMI. The prednisolone was used as standard. Plates were then incubated for 72 h at 37 °C in 5% CO_2_ incubator. After 72 h, cultures were pulsed with 25 μL of 0.5 μCi/well ^3^H-thymidine [Hartmann Analytic, Braunschweig, Germany], and plates were further incubated for 18 h. After incubation cells were harvested using a glass fibre filter and cell harvester [Inotech, Dottikon, Switzerland]. The level of the ^3^H-thymidine incorporated into the cells was measured by a LS65000 liquid scintillation counter [Beckman coulter, Fullerton, CA, USA]. Results were expressed as mean count per minutes (CPM). The 50% inhibitory concentration (IC_50_) values were calculated for all tested compounds.

#### Cytotoxicity assay

Cytotoxicity of test compounds on NIH-3T3 fibroblast cells (ATCC, Manassas, VA, USA) was evaluated by using the standard MTT colorimetric assay. Briefly 100 μL of 5 × 10^4^ cells/mL in Dulbacco’s modified eagles medium (DMEM) supplemented with 10% foetal bovine serum (FBS) were plated into 96-wells flat bottom plate and incubated overnight at 37 °C in 5% CO_2_. Three different concentrations of test compound (1, 10 and 100 µg/mL) were added to the plate in triplicates and incubated for 48 h. 0.5 mg/mL MTT (50 µL) was added to each well, the plate was then further incubated for 4 h. MTT was aspirated and 100 µL of dimethyl sulfoxide (DMSO) was then added to each well. The extent of MTT reduction to formazan within cells was calculated by measuring the absorbance at 540 nm, using spectrophotometer (Spectra Max plus, Molecular Devices, CA, USA). The cytotoxic activity was recorded as concentration causing 50% growth inhibition (IC_50_) for 3T3 cells. Cycloheximide was used as standard drug.

#### Statistical analysis

All data are reported as mean ± SD of the mean and the IC_50_ values were calculated using Excel based program. One-way ANOVA: *Post hoc* Dunnett test was also used and *p* < 0.05 was considered to indicate a statistically significant difference.

## Results and discussion

Purification of the methanol extract of the root-bark of *C. arborea* using various chromatographic techniques yielded compounds **1**–**5** (Figure 1). This is the first report of the isolation of compounds **1**, **2**, **3** and **4** from the genus *Cussonia*. Only compound **5** was previously isolated from *C. natalensis* (Fourie et al. 1989).

The EIMS of compound **1** showed a weak molecular ion peak at *m/z* 472.3547 (calcd. 472.3554), corresponding to molecular formula C_30_H_48_O_4_ in HR EIMS. The base peak at *m/z* 248, produced through retro-Diels-Alder fragmentation, and further loss of the carboxylic group led to the peak at *m/z* 203. The IR spectrum of compound **1** showed absorption of hydroxyl (3453 cm^−1^) and carbonyl (1699 cm^−1^) groups. The ^1^H-NMR spectrum exhibited signals due to 6 methyl singlets (δ 0.69, 0.97, 0.81, 1.12, 0.9 and 0.93, 3H each), an olefinic proton (δ 5.21, H-12), a hydroxyl-methine group (δ 3.57, H-3), one proton doublet of doublet at δ 2.83 (H-18) and a methylene attached to oxygen (δ 3.5, *J* = 10.8, H-23). The rest of signals were the sp^3^ CH and CH_2_ unit found at upfield region. The broad-band decoupled ^13 ^C-NMR (Table 1) and DEPT spectra displayed resonances for thirty carbons including 6 methyl, 11 methylene, 5 methine and 8 quaternary carbons. The most downfield peak at δ 181.87 was assigned to the carbonyl group of the acid (C-28). The spectra data also supported presence of a double bond (δ 123 and 145 ppm for C-12 and C-13, respectively) diagnostic signal for olean-12-enes (Begum et al. 2002). The structure of compound **1** was finally established as 3, 23-dihydroxyl-12-oleanen-28-oic acid (hederagenin) by comparison with existing literature (He et al. 2003).

Compound **2** has a molecular formula C_30_H_48_O_3_ deduced from HR EIMS with molecular ion at *m/z* 456.3566 (calcd. 456.3605) consistent with 7 degree of unsaturation. The base peak at *m/z* 248 and the fragment ion at *m/z* 203 showed a characteristic for a pentacyclic triterpene of β-amyrin series with a double bond between C-12 and C-13. The ^1^H-NMR spectrum showed 7 tertiary methyl groups at δ 0.96 (H-23), 0.75 (H-24), 0.88 (H-25), 0.73 (H-26), 1.11 (H-27), 0.88 (H-29) and 0.89 (H-30) on an oleanane skeleton. One proton doublet of doublet at δ 2.78 and a singlet olefinic proton at δ 5.26 were assigned to H-18 and H-12, indicating an olea-12-ene skeleton. The ^1^H-NMR spectrum also showed a deshielded signal for methine proton δ 3.18 (1H, t), which was assigned for H-3 proton. The ^13 ^C-NMR spectrum of compound **2** indicated the presence of 30 carbon atoms: 7 methyl, 10 methylene, 5 methine, and 8 quaternary carbons. The presence of oxygenated carbon at C-3 showed resonance at δ 78.9. The signal at δ 183.13 was due to carbon of carboxylic acid at C-28. On the basis of the spectra data (Table 2) and comparison of ^13 ^C shifts with the reported data, the structure has been identified as 3β-hydroxylolean-12-en-28-oic and commonly known as oleanolic acid (Mahato and Kundu 1994; Senthilkumar and Reetha, 2011).

Compound **3** showed molecular ion peak as well as base peak at *m/z* 412.3700 corresponding to molecular formula C_29_H_48_O (calcd. 412.3707) in the HR EIMS. In the ^1^H-NMR, 6 methyls appeared at δ 0.67 (H-18), 0.99 (H-19), 1.01 (H-21), 0.82 (H-26), 0.76 (H-27) and 0.78 (H-29). Three olefinic signals of 1 proton each were observed at δ 5.10 (dd, *J* = 15.2, 8.4 Hz, H-22), δ 4.96 (dd, *J* = 15.2, 8.8 Hz, H-23) and 5.32 (m, H-6) and their corresponding carbons resonated at δc 138.3, 129.3 and 121.7, respectively, which signified the presence of two double bonds in the compound. One proton signal at δ 3.46 ppm belongs to methine at H-3 (71.81 ppm) revealed that hydroxyl function was attached to it. This proton H-3 was coupled to methylene protons at H-2 (2.21 ppm), and this correlation between H-3 and H-2 was established by COSY analysis. The ^13 ^C-NMR revealed 29 signals, which were resolved using DEPT experiments into 6 methyl, 9 methylene, 11 methine, and 3 quaternary carbons. The spectra data (Table 3) of compound **3** were in good agreement with one reported for stigmasterol (Forgo and Kövér 2004).

Compound **4** was isolated as a white amorphous compound from fraction F_37_ (80% EtOAc in Hex). The ^1^H NMR spectrum showed signals for 6 methyl groups: 2 tertiary, 3 secondary, and 1 primary at δ 0.64 (H-18), 0.93 (H-19), 1.06 (H-21), 0.86 × 2 (H-26 & 27) and 0.88 (H-29), as well as 3 olefinic protons at 5.34 (H-6), 5.18 (H-22) and 5.02 (H-23) and an anomeric proton at 5.05 (d, *J* = 7.8 Hz), which were features of a triterpene glycoside. The ^13 ^C NMR spectrum of compound **4** was similar to that of **3** except for the signals that appeared between δ 70 and 80 region associated with sugar moiety. The anomeric carbon signal appeared at 102.59 and its proton at 5.05 ppm appearing as a doublet with coupling constant of 7.8 Hz. The most downfield peak at δ 140.94 was assigned to olefinic bonded quaternary carbon C-5. In HMBC spectrum H-1 of glc at δ of 5.05 exhibited a long range correlation with C-2 of aglycone at δ 32.12 and C-2 of glc at δ 78.10. Also, HMBC correlation was observed between H-23 of the aglycone at δ_H_ 5.02 and C-22 at δ 138.85. The FABMS of **4** (negative-ion mode) gave peak at *m/z* 573[M-H]^−^, indicating a molecular weight of 574 (calcd. 574.4235). The EIMS of **4** showed ion peaks at *m/z* 412, 394, 381, 351 and 255, which indicated that the aglycone is a stigmasterol. Compound **4** was characterized to be 3-*O*-β-d-glucopyranosyl stigmasterol on the basis of the spectra data (Table 4) as well as direct comparison (co-TLC) with authentic sample.

Compound **5** was obtained as a colourless solid. Its IR spectrum exhibited absorptions at 3453 (OH) and 1699 cm^−1^ (C = O). The positive-ion HR EIMS of compound **5** showed a molecular ion peak at *m/z* 470.3421, corresponding to C_30_H_46_O_4._ The peaks at *m/z* 248 (100%) and 203 (73%) supported the characteristics retro-Diels-Alder cleavage of Δ^12^-pentacyclic triterpenoid compound. The ^1^H NMR spectrum displayed signals due to 4 tertiary methyl groups at δ 0.77, 0.87, 1.06 and 1.23 (corresponding to position H-24, H-25, H-26, H-27, respectively) and 2 secondary methyl groups at δ 0.83 (d, 6.5 Hz, H-29) and 0.92 (d, 6.5 Hz, H-30). These characteristics together with the singlet olefinic proton at δ 5.23 ruled out an oleanane skeleton and confirmed the ursane framework (Table 5). Careful spectra studies and extensive review of the chemical literature confirmed compound **5** to be 23-hydroxy-3-oxo-urs-12-en-28-oic acid (Fourie et al. 1989).

**Table 5.** ^1^H NMR data of Compound **5** (500 MHz, CDCl_3_) compared with ^1^H NMR data of literature.

Position	^1^H δ (multiplicity, J)	^1^H* δ (multiplicity, J)
1.	1.27 (m)	
2.	2.00 (m)	
3.	–	–
4.	–	–
5.		
6.		
7.		
8.	–	–
9.		
10.	–	–
11.	1.90 (m)	
12.	5.23 (s)	5.23 (s)
13.	–	–
14.	–	–
15.		
16.		
17.	–	–
18.	2.15 (d, 11.5 Hz)	
19.		
20.		
21.		
22.		
23.	3.40 (d, 10.5 Hz) 3.70 (d, 10.5 Hz)	3.40 (d, 11.3 Hz) 3.62 (d, 11.3 Hz)
24.	0.77 (s)	0.82
25.	0.87 (s)	0.98
26.	1.06 (s)	1.10
27.	1.23 (s)	1.12
28.	–	–
29.	0.83 (d, 6.5 Hz)	0.83 (d, 6.4 Hz)
30.	0.92 (d, 6.5 Hz)	0.93 (d, 6.2 Hz)

* Fourie et al. (1989).

## Biological activity

**Figure 2.**
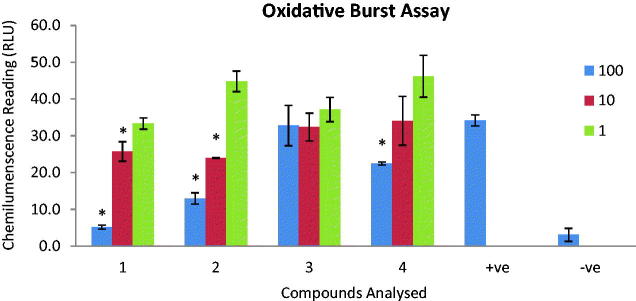
The graph represents the effect of compounds **1**–**4** on oxidative burst. Compounds were tested on three different concentrations (1, 10 and 100 µg/mL). Results are presented in relative light units (RLU) and oxidative burst activity of whole blood using luminol as a probe. Each vertical bar represents a mean of triplicate. Error bars represent standard deviations of the means. Significance difference was calculated using one-way ANOVA and * represent *p* < 0.05 significance difference was compared to the + ve control. Where + ve = cells + zymosan and − ve = cells alone.

**Figure 3.**
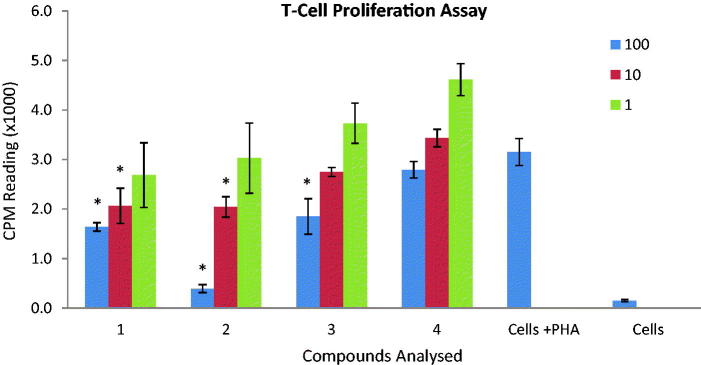
Effect of compounds on T-cell proliferation. Compounds were tested on three different concentrations (1, 10 and 100 µg/mL). Results are presented in counts per minutes (CPM). Each vertical bar represents a mean of triplicate. Error bars represent standard deviations of the means. Significance difference was calculated using one-way ANOVA. Significance difference was compared to the control having cells in the presence of PHA. Where * represent *p* < 0.05.

The immunomodulatory activities of compounds **1**–**4** were evaluated on two important parameters of innate and adaptive immune response, that is, the effect of compounds on production of intracellular ROS from serum opsonized zymosan activated whole blood phagocytes by luminol enhanced chemiluminescence technique (Figure 2) and inhibition of PHA induced human peripheral blood T-cells proliferation by radioactive thymidine incorporation (Figure 3). The compounds were also evaluated for their toxicity on NIH-3T3, normal fibroblast cells through MTT assay.

The data collected revealed that among all tested compounds (**1**–**4**), compounds **1** and **2** inhibited the production of ROS with an IC_50_ =  24.4 ± 4.3 and 37.5 ± 0.1 µg/mL, respectively, whereas compounds **3** and **4** did not inhibit the production of ROS (Table 6). The drug Ibuprofen was used as standard (IC_50_ = 11.2 ± 1.9 µg/mL). Compound **2** also exhibited marked inhibition of T-cell proliferation IC_50_ =  12.6 ± 0.4 µg/mL among others which either showed low level of activity (compound **3** with an IC_50_ =  86.8 ± 0.1 µg/mL) or no inhibition (compounds **1** and **4**), indicating good immunomodulatory potential of compound **2** (Table 6, Figure 3). However, the effect of these compounds on T-cell activation is much lower when compared with the standard drug used in this study while using the PHA activator. The steroidal immunosuppressive drug prednisolone was used as standard drug which showed 64.3 ± 2% inhibition at lowest tested concentration (0.62 µg/mL) in our laboratory. In a similar work, Magee and his co-workers (Magee et al. 2002) reported prednisolone with an IC_50_ value of 38.8 ng/mL. All compounds were found to be nontoxic on NIH-3T3 cells (Table 6). Inhibition of T-cell proliferation can serve as an approach to treat various immune disorders, including organ rejection after transplant (Khan et al. 2012). T-lymphocytes play an important role in the adaptive immunity by releasing various cytokines and enhancing the function of other immune cells including B-cells and macrophages (Mesaik et al. 2009; Khan et al. 2012; Mustafa et al. 2012). Compound **2** is a pentacyclic triterpenoid. The pentacyclic triterpenoids are a class of C_30_ isoprenoid compounds occurring widely in plants. Folding and cyclization of squalene leads to the dammarenyl ring system, which has a slightly different stereochemistry and ring structure from that of the major sterols (Dewick 2009). Their cytotoxic and anti-inflammatory activities have been reported in several studies (Neto 2011). Our results corresponds to reports by Ayatollahi et al. (2011) which documented inhibition of T-cell proliferation by pentacyclic triterpenes isolated from *Euphorbia microsciadia* Boiss (Euphorbiaceae) and they also proposed a mechanism by which pentacyclic triterpenoids could bring about this effect. They stated that the combination of E ring size as well as C-19, C-20 and C-28 positions could be responsible for the differences in biological effects in pentacyclic triterpenes analogues. However, in this study, hederagenin (**1**), a pentacyclic triterpenoid showed no inhibition. Thus, methylene oxide at position 4 could be a major factor, if not the only one, causing no antiproliferative activity observed in compound **1**. Compound **5** was not tested because the quantity isolated was not sufficient for the assay.

**Table 6.** Effect of pure compounds from *C. arborea* on phagocytes oxidative burst, T-cell proliferation and cytotoxicity on NIH-3T3 cells. The IC_50_ (µg / mL) was calculated using three doses (1, 10 and 100 µg/mL) of each compound. Values are expressed as mean ± SD of three determinations.

Compounds	ROS inhibition (IC_50_± SD µg/mL)	T-cell proliferation inhibition(IC_50_± SD µg/mL)	Cytotoxicity on NIH 3T3-cells(IC_50_± SD µg/mL)
**1**	24.4 ± 4.3	>100	>100
**2**	37.5 ± 0.1	12.6 ± 0.4	>100
**3**	>100	86.8 ± 0.1	>100
**4**	>100	>100	>100
**5**	NT	NT	NT
Ibuprofen	11.2 ± 1.9	–	–
Prednisolone	–	<0.62	–
Cyclohexamide	–	–	0.13 ± 0.02

NT means not tested.

## Conclusions

The present phytochemical investigation of root-bark of *C. arborea*, resulted in isolation of a total of three pentacyclic triterpenoids, a steroid and a steroidal glycoside. Including previous studies, 7 triterpenoids have been yielded from the title plant and 15 from the genus. These results may lead to the conclusion that the triterpenoids are the main constituents of the genus *Cussonia*. The immunomodulatory properties of its secondary metabolites (**1**–**4**) are being reported and results obtained suggests that compound **2** may be a potential therapeutic agent in treatment of various immune disorders, including organ rejection after transplant. Further studies are suggested on making new derivatives of **2** which could improve and enhance the immunomodulatory potential of compound **2**.
